# Beyond the Usual Suspects: Emerging *Pseudomonas* Species in Clinical and Environmental Niches

**DOI:** 10.3390/ijms27146210

**Published:** 2026-07-11

**Authors:** Andrea Marino, Stefano Stracquadanio, Federica Cosentino, Mariagiovanna Coco, Luigi La Via, Alessandro Franzò, Serena Spampinato, Emmanuele Venanzi Rullo, Antonino Maniaci, Giuseppe Nunnari

**Affiliations:** 1 Department of Clinical and Experimental Medicine, Infectious Diseases Unit, ARNAS Garibaldi Hospital, University of Catania, 95122 Catania, Italy; andrea.marino@unict.it (A.M.); federicacosentino91@gmail.com (F.C.); mariagiovannacoco@gmail.com (M.C.); alefranzosr@gmail.com (A.F.); serenaspampinato93@gmail.com (S.S.); giuseppe.nunnari1@unict.it (G.N.); 2 Department of Medicine and Surgery, University of Enna “Kore”, 94100 Enna, Italy; antonino.maniaci@unikore.it; 3 Department of General Surgery and Medical-Surgical Specialties (CHIRMED), University of Catania, 95123 Catania, Italy; luigilavia7@gmail.com; 4 Unit of Infectious Diseases, G. Martino University Hospital, Department of Clinical and Experimental Medicine, University of Messina, 98125 Messina, Italy; emmanuele.venanzirullo@unime.it

**Keywords:** *Pseudomonas*, emerging pathogens, non-aeruginosa, genomics, diagnostic challenges, bioremediation, One Health, antimicrobial resistance

## Abstract

Non-aeruginosa *Pseudomonas* (NAP) species represent a diverse and ubiquitous group of Gram-negative bacteria inhabiting a wide range of environmental niches, from soil and water to plant rhizospheres and clinical settings. While *Pseudomonas aeruginosa* has historically dominated clinical and research focus, the significance of NAP species, such as *Pseudomonas fluorescens*, *Pseudomonas putida*, and *Pseudomonas stutzeri*, as both opportunistic human pathogens and versatile biotechnological agents is increasingly recognized. Their remarkable genomic plasticity, driven by large accessory genomes and mobile genetic elements, underpins their metabolic versatility and adaptability but also facilitates the acquisition of virulence determinants and antibiotic resistance genes, contributing to their emergence in healthcare settings, particularly among immunocompromised individuals. This review provides a comprehensive analysis of NAP species, focusing on recent advances in their taxonomy facilitated by genomic tools like Whole-Genome Sequencing (WGS) and Multilocus Sequence Typing (MLST), which reveal complex species groups and challenge traditional classifications. We delve into the genomic landscape, exploring pangenome dynamics, horizontal gene transfer (HGT), and the genomic signatures that may differentiate clinical from environmental isolates. The clinical relevance of NAPs is examined, detailing the spectrum of infections, epidemiological trends, risk factors, and insights into virulence mechanisms, including secretion systems (T3SS, T6SS) and pathogenicity islands. Addressing a critical need, this review incorporates detailed sections on the diagnostic challenges posed by NAPs, including common misidentifications and the role of modern techniques like MALDI-TOF MS and WGS, and outlines current and novel therapeutic strategies, considering the growing problem of antimicrobial resistance (AMR) within this group. Furthermore, the biotechnological applications of NAPs in bioremediation and biocatalysis are discussed alongside evolving biosafety considerations, reflecting the shift from strict containment to integrated monitoring approaches for genetically engineered strains. By synthesizing current knowledge and highlighting research gaps, this review underscores the necessity of integrated, One Health approaches to understand and manage the dual nature of non-aeruginosa *Pseudomonas* species as both environmental inhabitants and clinically relevant pathogens.

## 1. Introduction

The genus *Pseudomonas* encompasses a vast and metabolically diverse group of Gram-negative bacteria, ubiquitous in terrestrial and aquatic environments [1]. Within this genus, *Pseudomonas aeruginosa* has long held the spotlight as a model organism and a formidable human opportunistic pathogen, responsible for a significant burden of healthcare-associated infections, particularly ventilator-associated pneumonia and sepsis syndromes, and notorious for its intrinsic and acquired multidrug resistance (MDR) [2]. This focus, while justified by the clinical impact of *P. aeruginosa*, has historically overshadowed the relevance of other members of the genus, collectively referred to as non-aeruginosa *Pseudomonas* (NAP) species. It should be emphasised at the outset that “non-aeruginosa *Pseudomonas*” is an operational, clinically motivated designation rather than a monophyletic or biologically cohesive taxon: it encompasses a highly heterogeneous assemblage of species distributed across several phylogenetic groups of the genus. Consequently, the generalisations offered here derive predominantly from the three best-characterised groups—the *P. fluorescens* complex, the *P. putida* group, and the *P. stutzeri*/*Stutzerimonas* complex—and should not be assumed to apply uniformly to all non-aeruginosa species.

However, the landscape of clinical microbiology is in continuous evolution, with a growing appreciation for the role of NAPs as significant opportunistic pathogens in their own right. It is crucial to recognize that the pathogenicity of certain NAPs is not a recent discovery. Early clinical reports dating back decades documented infections caused by species such as *P. fluorescens* and *P. putida*, demonstrating their capacity to cause serious illness, including sepsis and meningitis, particularly in vulnerable patient populations [3]. Reviews, such as the one by Scales et al. (2014), have previously highlighted the clinical significance of the *P. fluorescens* species complex [4]. Therefore, the current attention towards NAPs should not be framed as the discovery of entirely “emerging” pathogens, but rather as an “increasing recognition” of their clinical impact. This renewed focus is driven by several converging factors: the advancement of diagnostic technologies, particularly matrix-assisted laser desorption ionization-time of flight mass spectrometry (MALDI-TOF MS) and whole-genome sequencing (WGS), which allow for more accurate and frequent identification of NAP species previously misidentified or grouped generically [5]; and the expansion of susceptible patient populations, including immunocompromised individuals (e.g., cancer patients, transplant recipients), patients with indwelling medical devices, and those undergoing invasive procedures [6]. Species such as *P. fluorescens*, *P. putida*, and *P. stutzeri*, once primarily studied for their environmental roles or considered mere contaminants, are now definitively linked to a range of human infections, including bacteremia, urinary tract infections (UTIs), skin and soft tissue infections (SSTIs), and respiratory infections [7,8,9,10].

This increasing recognition is reinforced by data from the current decade. Genome-based taxonomy has continued to reshape the group: the *P. stutzeri* complex was reclassified into the new genus *Stutzerimonas* in 2022 [11], and a 2024 pangenomic analysis of the *P. putida* group refined its internal structure and catalogued its resistance determinants [12]. Contemporary genomic surveillance has begun to document NAP as clinically meaningful reservoirs of carbapenem resistance: a whole-genome-sequencing study conducted in 2022–2024 in a Spanish tertiary hospital characterised carbapenemase (predominantly metallo-β-lactamase)-producing isolates spanning eight species of the *P. putida* group and *Stutzerimonas*, with resistance exceeding 55% to β-lactams, aminoglycosides and fluoroquinolones and susceptibility largely retained only to cefiderocol, amikacin and colistin [13]. Together with the population-based rise in *Stutzerimonas* (*P. stutzeri*) bloodstream infection reported from Queensland [14] and the continued recovery of newly described species from clinical specimens (e.g., *P. kurunegalensis* from urine [15]), these observations justify a focused, up-to-date synthesis of the group.

A unifying characteristic underpinning the adaptability and diverse lifestyles of NAP species is their remarkable genomic plasticity. While *P. aeruginosa* is also known for its dynamic genome [16], this trait is profoundly evident across the NAP spectrum and is fundamental to understanding its ecological success and opportunistic pathogenicity. NAP genomes are characterized by a relatively conserved core genome, encoding essential housekeeping functions, complemented by a large and variable accessory genome [17]. This accessory genome is a repository of genes acquired through horizontal gene transfer (HGT), often mediated by mobile genetic elements (MGEs) such as plasmids, transposons, integrons, and bacteriophages [18].

This genomic fluidity confers significant advantages. It allows NAP populations to rapidly adapt to fluctuating environmental conditions, exploit novel nutrient sources, and resist various stresses, including exposure to pollutants or antimicrobial agents [19,20]. The same mechanisms that enable *P. putida* KT2440 to degrade aromatic hydrocarbons or tolerate organic solvents, making it a valuable chassis for bioremediation and biotechnology [21,22], can also facilitate the acquisition and dissemination of antibiotic resistance genes (ARGs) and virulence factors in clinical settings [23]. Therefore, genomic plasticity is a double-edged sword, especially within the One Health frame: it is the foundation of their beneficial applications but also the engine driving their potential as opportunistic pathogens capable of acquiring traits necessary for host colonization, immune evasion, and resistance to therapy. Understanding the mechanisms and consequences of this plasticity is central to both harnessing NAPs for biotechnology and mitigating the risks they pose in healthcare environments.

This review aims to provide a comprehensive and critical synthesis of the current understanding of NAP species, moving beyond the shadow of *P. aeruginosa*. We focus primarily on three well-studied and clinically relevant species—*P. fluorescens*, *P. putida*, and *P. stutzeri*—as exemplars, while also incorporating information on other NAPs where relevant. The objectives are to (i) explore the evolving taxonomy and complex genomic landscape of NAPs, highlighting the impact of modern sequencing technologies; (ii) critically evaluate their clinical significance, detailing the spectrum of infections, epidemiology, risk factors, and underlying virulence mechanisms, addressing the need for more mechanistic insights; (iii) provide a detailed overview of diagnostic challenges and advances, including methods for accurate identification and differentiation; (iv) present a thorough analysis of therapeutic strategies, encompassing both established and novel antimicrobial agents and the growing challenge of antimicrobial resistance (AMR); (v) discuss their established and potential roles in environmental and biotechnological applications, including updated perspectives on biosafety; and (vi) identify key knowledge gaps and propose future research directions, integrating a One Health perspective.

## 2. Taxonomy and Genomic Landscape of NAP

The genus *Pseudomonas* is notoriously complex and diverse, and its taxonomy has undergone significant revisions as molecular methods have superseded traditional phenotypic characterization [24,25]. Accurately delineating species, particularly within the NAP groups, is crucial for understanding their ecology, pathogenic potential, and biotechnological utility.

### 2.1. Current Taxonomic Classification: Integrating Whole-Genome Sequencing (WGS) and Multilocus Sequence Typing (MLST)

Historically, the classification of *Pseudomonas* species relied heavily on phenotypic tests, such as growth characteristics, biochemical reactions (e.g., oxidase test, pigment production, growth at 42 °C), which are still useful for initial identification of isolates like *P. aeruginosa* [26]. However, these methods often lack the resolution to reliably differentiate the many closely related species within the NAP groups [24,25]. The advent of molecular techniques, initially 16S rRNA gene sequencing, provided a phylogenetic framework but often failed to resolve species within highly related complexes due to the gene’s conserved nature [27].

A paradigm shift occurred with the widespread adoption of Multilocus Sequence Typing (MLST), which analyzes sequence variation in several housekeeping genes (e.g., *gyrB*, *rpoB*, *rpoD*), and more recently, Whole-Genome Sequencing (WGS) [28,29]. WGS provides the ultimate resolution, allowing for comprehensive comparisons based on overall genome relatedness indices like Average Nucleotide Identity (ANI) and digital DNA–DNA hybridization (dDDH) [29]. These genomic approaches have revealed that many groups previously considered single species are, in fact, species complexes comprising multiple distinct lineages or genomovars (genomic species) [24,29].

- *P. fluorescens* complex: Originally defined broadly, genomic analyses have shown this group comprises numerous distinct species, many reclassified from isolates initially labelled *P. fluorescens* [20]. MLSA and phylogenomic studies have divided the complex into several subgroups (e.g., *P. corrugata*, *P. chlororaphis*, *P. fluorescens* sensu stricto) [25,29,30].
- *P. putida* group: Similarly, WGS and ANI analyses indicate that strains historically classified as *P. putida* are phylogenomically diverse and dispersed across a wider evolutionary group that includes other named species [31]. Many clinical isolates previously identified as *P. putida* by biochemical methods have been reclassified as distinct species (e.g., *P. monteilii*, *P. asiatica*, or novel species) upon WGS analysis [32].
- *P. stutzeri* complex: This non-fluorescent group is also genomically diverse, comprising numerous genomovars defined by DNA–DNA hybridization and now corroborated by ANI and dDDH thresholds. WGS analysis has identified dozens of distinct genomovars within the complex, including previously unknown lineages [33,34].

WGS is now considered the gold standard for definitive *Pseudomonas* species identification, resolving ambiguities left by other methods and revealing the true extent of diversity. However, its routine implementation in clinical labs faces challenges related to cost, turnaround time, and bioinformatics expertise. MLST remains a valuable tool for phylogenetic placement and epidemiological tracking, offering better resolution than 16S rRNA sequencing alone [35]. The ongoing refinement of *Pseudomonas* taxonomy using genomic data is essential for accurate diagnosis, appropriate treatment selection, and reliable risk assessment.

These taxonomic refinements are not merely academic; accurate species assignment has direct clinical and epidemiological consequences. First, for epidemiology and burden estimation, whole-genome-sequencing-based re-identification repeatedly shows that isolates reported biochemically as *P. putida* or *P. fluorescens* in fact comprise many distinct species. A recent genomic study of carbapenemase-producing NAP (2022–2024) resolved such a collection into eight species across the *P. putida* group and *Stutzerimonas*, including *P. alloputida*, *P. mosselii* and *P. kurunegalensis* [13]. Second, for outbreak investigation, the resolution of MLST and WGS distinguishes genuine clonal transmission from the coincidental clustering of unrelated environmental strains. Third, for therapy, because intrinsic resistance and pharmacokinetic/pharmacodynamic behaviour can differ between species, precise identification is a prerequisite for the eventual establishment of species-specific breakpoints (Section 5). Fourth, genome-based taxonomy continually uncovers clinically relevant species: a consequential example is the 2022 reclassification of the *P. stutzeri* complex into the genus *Stutzerimonas* [11,36], so that much of the clinically relevant *P. stutzeri* literature now maps onto *Stutzerimonas stutzeri* and related species. For continuity with the cited literature, we retain the familiar *P. stutzeri* usage below while signposting the current *Stutzerimonas* nomenclature.

### 2.2. Pangenome Dynamics and Accessory Genome: Drivers of Diversity

The genomic diversity within NAP species complexes is reflected in their pangenome structures. The pangenome represents the entire gene repertoire of a species or group, consisting of the core genome (genes present in all or nearly all strains), the accessory genome (genes present in some but not all strains), and unique genes (found in single strains) [37].

Studies on NAP groups consistently reveal large accessory genomes and relatively small core genomes, indicating significant genomic fluidity and heterogeneity [17].

- The *P. fluorescens* complex, based on 93 genomes, was found to have a core genome of only 1334 coding sequences (CDSs) but a vast pangenome of 30,848 CDSs, suggesting an “open” pangenome (i.e., one whose total gene repertoire keeps expanding as further genomes are sequenced—reflecting frequent, largely HGT-mediated gene gain—as opposed to a “closed” pangenome, which plateaus once a modest number of genomes has been sampled) where sequencing additional strains continues to add novel genes [17].
- The *P. stutzeri* complex (123 genomes) exhibits a core genome of ~1100 gene clusters and an open pangenome of over 13,000 gene families, with a large proportion (~81%) being accessory genes. Functional annotation is lacking for a significant portion (~45%) of these pangenome genes [33].
- The *P. putida* group (413 genomes) possesses a core genome of 2226 protein families, but its pangenome contains over 77,000 distinct protein families, again highlighting immense diversity [11].

This genomic structure contrasts with species like *P. aeruginosa*, which, while adaptable, tends to have a larger core genome relative to its pangenome, suggesting a more conserved evolutionary trajectory within the species, although its accessory genome is still dynamic [10,11,17,38].

The large accessory genome is the primary driver of phenotypic diversity and niche adaptation in NAPs. It harbours genes involved in specialized metabolic pathways (e.g., degradation of xenobiotics), secondary metabolite production (e.g., antibiotics, siderophores), stress resistance, and interactions with hosts or competitors, including virulence factors and ARGs [30,39]. The dynamic nature of the accessory genome, shaped by gene gain and loss events often mediated by MGEs, allows NAP populations to rapidly evolve and adapt to new challenges and opportunities in diverse environments, including the human host [30,39].

### 2.3. MGE and HGT in NAP

HGT, the movement of genetic material between organisms other than by vertical descent, is a major force shaping the accessory genome and driving the evolution of NAPs [39]. MGEs are the primary vehicles for HGT in bacteria.

- Plasmids: Plasmids play a critical role in the rapid dissemination of AMR in clinical settings. For example, a multidrug-resistant *P. stutzeri* isolate from cerebrospinal fluid was found to carry the carbapenemase gene *blaVIM-2* on a novel conjugative plasmid, pZDHY95-VIM-2 [40]. While less extensively studied in all NAPs, plasmids are undoubtedly key players in their adaptation. Experimental transfer of plasmids like the TOL plasmid between *P. putida* and *P. aeruginosa* has been demonstrated [41,42].
- Transposons and Insertion Sequences (IS Elements): IS elements are frequently found flanking resistance genes or integrated into plasmids and integrons, facilitating the mobilization and rearrangement of these genes. Studies on clinical *P. putida* isolates have shown that genes associated with survival in the host (e.g., oxidative stress resistance, biocide resistance) are often located on transposons, distinguishing them from environmental strains [43].
- Integrons: Genetic platforms that capture and express gene cassettes, typically encoding ARGs. Class 1 integrons are particularly prevalent on plasmids and transposons in Gram-negative bacteria and are strongly associated with MDR. The *blaVIM-2* gene found in the *P. stutzeri* plasmid pZDHY95-VIM-2 was located within a class 1 integron [40]. Similarly, integrons carrying various resistance cassettes have been identified in clinical *P. putida* isolates harboring *blaVIM-2* [44,45].
- Outer Membrane Vesicles (OMVs): Recent research, primarily in *P. aeruginosa*, has shown that OMVs, naturally released vesicles containing proteins, lipids, and nucleic acids, can package and transfer plasmid DNA, including ARGs, between bacteria. This mechanism protects the DNA from environmental degradation and can facilitate HGT, particularly within biofilms [46]. While direct evidence in NAPs is limited, OMV-mediated HGT is a plausible mechanism contributing to gene exchange within these species as well. We explicitly flag this as a high-priority knowledge gap: whether OMV-mediated transfer moves resistance determinants between *P. aeruginosa* and co-resident NAPs within dense polymicrobial biofilms—such as those of the cystic fibrosis airway or chronic wounds—remains essentially untested, and represents a plausible but currently unquantified route for the dissemination of carbapenemase genes.

The abundance and activity of these MGEs underscore the high potential for NAPs to acquire and exchange genetic information, contributing significantly to their genomic plasticity and rapid adaptation. The functional importance of MGEs in conferring adaptive traits like AMR in specific NAP strains (e.g., *P. stutzeri*, clinical *P. putida*) is well-supported.

Given the clinical prominence of *P. aeruginosa* and its frequent co-occurrence with NAPs in various environments (soil, water, hospital settings, polymicrobial infections) [47], the potential for HGT between *P. aeruginosa* and NAP species is a significant concern, particularly regarding the spread of virulence and resistance genes.

Direct evidence for the transfer of comprehensive ARG-harboring MGEs from *P. aeruginosa* to NAPs in clinical settings appears limited based on current studies. A study investigating *blaVIM*-positive *P. putida* group strains and *P. aeruginosa* isolates from the same hospital environment found that while both species carried *blaVIM-2*, there was no evidence of exchange of the larger MGEs carrying these genes between the two species during the study period, suggesting separate acquisition events or limited inter-species transfer of these specific elements in that context [48].

However, the potential for transfer exists:

- Shared MGEs and Genes: The presence of identical or highly similar resistance genes (like *blaVIM-2*) and MGEs (like specific class 1 integrons) in both *P. aeruginosa* and certain NAPs (e.g., *P. stutzeri*) strongly implies that HGT occurs, although determining the direction and frequency can be challenging.
- Experimental Evidence: Laboratory studies have demonstrated the possibility of conjugal plasmid transfer between *Pseudomonas* species, such as the transfer of the TOL plasmid from *P. putida* to *P. aeruginosa* [42].
- Ecological Opportunity: Co-colonization in niches like the gut of hospitalized patients, chronic wounds, or the lungs of cystic fibrosis patients provides ample opportunity for genetic exchange. Environmental reservoirs also serve as potential melting pots for HGT [39,49].

Therefore, while large-scale, frequent transfer of complex resistance or virulence islands directly from *P. aeruginosa* to NAPs might not be the dominant mode of adaptation for all NAPs, the exchange of specific genes or smaller MGEs is plausible and likely contributes to the evolution of clinically relevant traits in NAP populations. NAPs themselves can also serve as reservoirs and potential donors of genes to *P. aeruginosa* [50]. The complex interplay and directionality of HGT within the *Pseudomonas* genus require further investigation.

### 2.4. Phylogenetic Diversity Within Key NAP Species (P. fluorescens, P. putida, P. stutzeri)

Beyond the species complex level, significant phylogenetic diversity exists within individual NAP species or genomovars. Understanding this intraspecific diversity is crucial for linking genotype to phenotype, particularly concerning pathogenic potential versus environmental adaptation.

A key question is whether clinical isolates associated with human infections represent distinct pathogenic sub-lineages that have diverged from their typically non-pathogenic environmental counterparts. Phylogenetic analyses based on MLST or WGS attempt to address this.

- *P. fluorescens* complex: Research suggests that virulence potential might be clade-specific within this complex. For instance, the presence of a functional T3SS, a key virulence determinant in many Gram-negative pathogens, has been reported to be specific to certain subclades of *P. fluorescens* [51]. This implies that only a subset of *P. fluorescens* complex strains may possess the genetic machinery typically associated with invasive pathogenesis. Further research correlating specific phylogenetic clades with clinical origin and virulence phenotypes is needed.
- *P. putida* group: Studies using MLSA based on nine housekeeping genes and ANI analysis on a collection of clinical and nonclinical *P. putida* group isolates found a mixed distribution. Clinical and nonclinical isolates were often interspersed within the same phylogenetic clusters or species-level ANI groups. This suggests that, at least based on core genome phylogeny, distinct “pathogenic lineages” may not be readily identifiable within the broader *P. putida* group. The observation that clinical and non-clinical isolates do not segregate into separate core-genome clades [11,31], together with the finding that host-survival-associated genes in clinical strains are frequently transposon-borne [48], is more consistent with pathogenic potential arising from HGT-mediated acquisition of accessory genes than from a trait fixed within a discrete ancestral sub-lineage. We present this as the most parsimonious reading of the available comparative-genomic data rather than as an established conclusion; direct functional evidence remains limited.
- *P. stutzeri* complex: Given its vast diversity and multiple genomovars isolated from diverse sources (including clinical), it is likely that significant phylogenetic structuring exists. However, studies explicitly correlating specific phylogenetic clades within the *P. stutzeri* complex with clinical origin or enhanced virulence are still limited. The study by Li et al. (2022) addresses the environmental origins and genomic diversity of the complex rather than the determinants of clinical infection [33]. We note only that, given the strong association of *P. stutzeri* infections with advanced age, comorbidity and healthcare exposure (Section 3.2), host factors are widely regarded as important predisposing determinants; the relative contribution of specific bacterial lineages versus host susceptibility has not been formally established and remains an open question.

Overall, the relationship between core genome phylogeny and pathogenic potential in NAPs appears complex and likely varies between species groups. While some groups like *P. fluorescens* might show clade-associated virulence potential, in others like *P. putida*, pathogenicity seems more related to the flexible accessory genome. Regardless of whether distinct pathogenic lineages exist, comparative genomics can identify specific genes or genomic features that are enriched in clinical isolates compared to environmental ones, potentially serving as genomic signatures of adaptation to the host environment [11,30,48].

- Accessory Genes: As mentioned, clinical *P. putida* isolates possess specific genes, often on transposons, related to survival under oxidative stress, biocide resistance, specific amino acid metabolism pathways, and toxin-antitoxin systems, which are largely absent in environmental isolates analysed in the same study [43]. These functions likely confer advantages for colonization and persistence within human tissues.
- Nutrient Acquisition: Clinical *P. putida* strain H8234 was found to possess unique TonB-dependent transporters for acquiring xenosiderophores (siderophores produced by other microbes), similar to those found in *P. aeruginosa*. This enhanced iron-scavenging capability could provide a significant fitness advantage in the iron-limited environment of the human host [52].
- Secretion Systems: The identification of a T3SS-like system in a clinical *P. putida* isolate suggests potential acquisition of virulence machinery associated with host cell manipulation [53].
- Resistance Genes: While ARGs can be found in environmental isolates (e.g., *P. fluorescens* from Antarctica harbouring efflux pump genes and even a carbapenemase gene) [54], clinical isolates, particularly those from hospital settings, often exhibit a higher prevalence and diversity of ARGs, especially those conferring resistance to clinically relevant antibiotics [55].
- Pathogenicity Islands (PAIs): Although classically defined PAIs might be less common or well-characterized in NAPs compared to canonical pathogens, specific genomic regions enriched in virulence-associated or host-adaptation genes exist. For example, Antarctic *P. fluorescens* isolates were found to harbor PAIs, though their specific contribution to potential virulence in that context is unclear [54]. The unique regions identified in clinical *P. putida* carrying adaptive genes might be considered functional equivalents of PAIs [56].

Identifying these genomic features is crucial for understanding the evolutionary pathways leading to opportunistic pathogenicity in NAPs and for developing diagnostic markers to assess the potential risk posed by specific isolates. However, it is important to note that the presence of “virulence-associated” genes does not automatically equate to pathogenicity, as their expression and function are context-dependent. Table 1 summarizes key comparative characteristics of the three focal NAP species complexes.

Two caveats must temper the interpretation of these observations. First, in the best-studied group (the *P. putida* group), clinical and environmental isolates are frequently interspersed within the same phylogenomic clusters, so distinct “pathogenic lineages” are not readily discernible from core-genome phylogeny [11,31,48]. Second, the accessory genes enriched in clinical collections are typically carried on mobile genetic elements, and are therefore better explained by independent HGT events than by stable evolutionary divergence toward a host-adapted lifestyle. Accordingly, enrichment of a given accessory gene in clinical isolates should be read as a candidate marker requiring functional and epidemiological validation, not as demonstration of adaptation to the human host or of a genetically distinct pathogenic sub-population.

## 3. Clinical Significance and Pathogenesis of NAP Infections

While often overshadowed by *P. aeruginosa*, NAP species are increasingly implicated in a diverse range of human infections, particularly in healthcare settings and among vulnerable patient populations. Understanding their clinical spectrum, epidemiology, and pathogenic mechanisms is crucial for effective diagnosis and management.

### 3.1. Spectrum of Infections Caused by NAP Species (P. fluorescens, P. putida, P. stutzeri, and Others)

NAP species can cause infections affecting various body sites, although the specific spectrum can differ between species:

- *P. fluorescens* complex: Historically considered non-pathogenic [3], this group is now recognized primarily as a cause of bacteraemia, often linked to iatrogenic sources. Outbreaks have been traced to contaminated blood products (due to the group’s ability to grow at refrigeration temperatures), intravenous fluids, saline flushes, and medical equipment [4,9]. Respiratory isolates are also reported, although their role as primary pulmonary pathogens is less clear than that of *P. aeruginosa*. An intriguing association exists between *P. fluorescens* (specifically antibodies against its I2 antigen) and Crohn’s disease, although a causal link remains unproven [85].
- *P. putida* group: These species are frequently associated with opportunistic infections in immunocompromised individuals or patients with breaches in host defences. Common manifestations include bacteraemia (often catheter-related), skin and soft tissue infections (SSTIs), particularly following trauma, burns, or in patients with chronic wounds or peripheral vascular disease [8,86]. Pneumonia and urinary tract infections are also reported. While generally considered low virulence, severe sepsis and fatal outcomes have been documented, especially in patients with significant comorbidities [7,87].
- *P. stutzeri* complex: This species is infrequently isolated from clinical material compared to other pseudomonads. When found, it most commonly originates from wounds and urine, often representing colonization rather than true infection. However, *P. stutzeri* can cause bacteraemia, particularly in elderly patients with chronic comorbidities. Ear infections (otitis) are also relatively common sources of isolation. Rarer invasive infections like pneumonia, meningitis, and endocarditis have been reported, typically in immunocompromised hosts or following surgery [88,89].

Other NAP species, such as *P. mendocina* [90], *P. alcaligenes* [91], *P. monteilii* [92], and *P. fulva* [93], have also been implicated in human infections, further highlighting the broad pathogenic potential within the genus beyond *P. aeruginosa*.

### 3.2. Prevalence, Epidemiology, and Risk Factors

Quantifying the true prevalence and burden of NAP infections is challenging due to historical misidentification and lack of systematic surveillance specifically targeting these species [76]. Unlike *P. aeruginosa*, for which extensive surveillance programs exist (e.g., SENTRY program) [94], global data for NAPs are scarce. Available data often come from single-center studies or outbreak investigations.

Studies suggest NAPs are relatively uncommon compared to *P. aeruginosa*. In a 10-year hospital study, *P. stutzeri* accounted for only 0.9% of *Pseudomonas* isolates [95]. In a study of cancer patients, NAPs (*P. putida*, *P. fluorescens*, *P. stutzeri*, *P. mendocina*) represented 104 cases over 10 years, considered rare but notable [6]. A population-based study in Queensland, Australia (2000–2019) identified 228 episodes of *P. stutzeri* bloodstream infection, noting an increasing incidence over time, particularly in older males [14]. *P. fluorescens* outbreaks linked to contaminated products can involve significant numbers of patients across multiple states.

NAP infections can be community-associated, healthcare-associated, or nosocomial. *P. stutzeri* bloodstream infections in Australia were predominantly community-onset (community-associated or ambulatory healthcare-associated) [14]. *P. fluorescens* outbreaks are often linked to contaminated compounded pharmaceuticals or blood products [80,81,82]. *P. putida* infections are frequently nosocomial, associated with medical devices or occurring in immunocompromised patients, but can also result from environmental exposure (e.g., contaminated water) [7,83]. Geographic variations may exist, with *P. stutzeri* BSI incidence in Queensland being higher in warmer, more humid regions and during rainy months [14]. There is currently limited direct evidence correlating large-scale industrial or environmental use of specific NAP strains with subsequent increases in clinical infections by those strains, although this remains a theoretical concern, especially regarding AMR dissemination.

Key risk factors for NAP infections mirror those for *P. aeruginosa* and include [96,97,98]:

- Being immunocompromised (e.g., hematologic malignancies, chemotherapy-induced neutropenia, HIV/AIDS, organ transplantation, steroid use);
- Indwelling medical devices (e.g., central venous catheters, urinary catheters, ventilators);
- Underlying chronic diseases (e.g., cancer, cystic fibrosis, diabetes mellitus, chronic kidney disease, peripheral vascular disease);
- Breaches in skin integrity (e.g., severe burns, surgical wounds, chronic ulcers, trauma, dermatitis);
- Extremes of age (neonates, elderly);
- Malnutrition and debilitation;
- Recent surgery or invasive procedures;
- Exposure to contaminated medical products or environments (e.g., contaminated IV fluids, blood products, hospital water sources, improperly maintained hot tubs).

Understanding these risk factors is essential for identifying patients vulnerable to NAP infections and implementing preventive measures.

### 3.3. Mechanistic Insights into NAP Virulence

While generally less virulent than *P. aeruginosa*, NAPs possess an array of factors that contribute to their ability to colonize hosts, evade immune defences, and cause disease, particularly under opportunistic conditions.

NAPs utilize various factors, some shared with *P. aeruginosa* and others potentially distinct, to interact with their hosts:

Adhesion and Motility: Adhesion and motility in non-aeruginosa *Pseudomonas* species are far from generic, showcasing specialized adaptations finely tuned to their diverse ecological niches. For instance, *Pseudomonas putida* often employs lophotrichous flagella (a tuft at one pole) for robust movement in complex environments like soil, while *Pseudomonas stutzeri* typically utilizes a single polar flagellum, efficient for navigating aquatic settings. Beyond swimming, Type IV pili (T4P) mediate twitching motility, allowing these bacteria to explore surfaces. Crucially, for attachment, large afimbrial adhesins such as LapA are pivotal in species like *Pseudomonas fluorescens* and *P. putida.* LapA facilitates strong, irreversible attachment to a variety of surfaces, including plant roots, plastic, and glass, which is a critical first step in colonization and biofilm formation. This initial attachment is often supported by other large adhesins, for example, LapF in *P. putida* contributes to cell-to-cell interactions and biofilm structure, and MapA in *P. fluorescens* aids in biofilm maturation. Type IV pili also function directly as adhesins; in *P. stutzeri*, they are vital not only for surface binding but also for processes like natural DNA uptake. These distinct molecular mechanisms are fundamental for these bacteria to effectively colonize their specific environments, ranging from the plant rhizosphere and food processing equipment to, occasionally, medical devices like catheters [57,99,100].

Enzymes and Toxins: Some NAPs produce extracellular enzymes that can damage host tissues or modulate immune responses. Certain *P. fluorescens* strains exhibit haemolytic activity, mediated by factors including phospholipase C and potentially T3SS effectors, contributing to red blood cell lysis and tissue damage [51]. Proteases and lipases may also play roles, although less characterized than in *P. aeruginosa* [101].

Siderophores: Iron acquisition is essential for bacterial survival in the host. The fluorescent *Pseudomonas*—including both *P. fluorescens* and *P. putida*—produce pyoverdine-type siderophores (fluorescent iron-chelating pigments with a conserved chromophore and strain-specific peptide chains) to scavenge iron under the iron-restricted conditions of the host [102]. Individual strains characteristically produce their own structural variant of pyoverdine. The ability of some clinical *P. putida* strains to utilize xenosiderophores via specific TonB-dependent transporters represents a potential adaptation enhancing survival in iron-limited host environments [52].

Bioactive Secondary Metabolites: Particularly within the *P. fluorescens* complex, strains produce diverse secondary metabolites (e.g., 2,4-diacetylphloroglucinol (DAPG), phenazines, pyrrolnitrin) primarily known for their roles in biocontrol against plant pathogens [59]. While their direct role in human infection is less clear, some metabolites could potentially modulate host immune responses or interact with host cells.

**Secretion Systems**

Secretion systems are sophisticated nanomachines used by Gram-negative bacteria to deliver effector proteins that manipulate host cells or compete with other microbes. Their presence and function in NAPs are areas of active investigation.

Type III Secretion System (T3SS): This system injects effector proteins directly into eukaryotic host cells, often subverting immune responses or inducing cytotoxicity.

∘ *P. fluorescens:* Functional T3SS gene clusters, homologous to those in plant pathogens like *P. syringae*, have been identified in several *P. fluorescens* strains, particularly those associated with plant roots. Some studies suggest T3SS presence is specific to certain phylogenetic subclades. Experimental evidence shows that T3SS in *P. fluorescens* can secrete effector proteins (e.g., Rop effectors) and, in some strains, contributes to interactions with host cells, such as haemolytic activity or virulence towards macrophages in vitro. Its direct role in human clinical infections requires further investigation, but its presence suggests a potential for host cell manipulation [51].
∘ *P. putida:* To date, a T3SS-like gene cluster has been reported in the genome of a single clinical *P. putida* isolate [53]; this remains an isolated genomic observation rather than established evidence of functional virulence machinery. However, functional data demonstrating T3SS activity and its contribution to virulence in clinical *P. putida* infections are currently limited.
∘ *P. stutzeri:* T3SS is generally not considered a typical feature associated with *P. stutzeri* pathogenicity in humans.

Type VI Secretion System (T6SS): This system delivers effector proteins into both prokaryotic and eukaryotic cells, playing roles in interbacterial competition and host cell interactions.

∘ *P. fluorescens:* Genomes within the *P. fluorescens* complex typically encode one or more T6SS clusters, likely involved primarily in competition within microbial communities [103].
∘ *P. putida:* Strains like KT2440 possess multiple T6SS clusters (K1, K2, K3). The K1-T6SS has been experimentally shown to be a potent antibacterial weapon, secreting toxic effectors (e.g., Tke2) that kill competing bacteria, including plant pathogens. This T6SS contributes to the biocontrol capabilities of *P. putida* and its fitness in polymicrobial environments [60]. A direct role in human infection has not been established.
∘ *P. stutzeri:* While T6SS loci are found in *Pseudomonas* species, specific studies detailing their role in *P. stutzeri* virulence are lacking. Given their role in interbacterial competition, they likely contribute to niche adaptation.

The presence of these secretion systems, particularly T3SS in certain *P. fluorescens* and potentially *P. putida* strains, indicates a capacity for more direct interaction with host cells than previously appreciated, contributing to their opportunistic pathogenic potential. T6SS likely plays a more significant role in niche competition and shaping polymicrobial interactions.

The ability to form biofilms—structured communities of bacteria encased in a self-produced matrix—is a key virulence trait for many bacterial pathogens, including *P. aeruginosa* [104]. NAPs, particularly *P. fluorescens*, are also known to readily form biofilms on various surfaces, including medical devices like catheters and environmental surfaces. Biofilm formation provides protection from host immune defenses (e.g., phagocytosis), antimicrobial agents, and environmental stresses. This capacity is critical for the persistence of NAPs in chronic infections and for their role in device-associated infections, such as the prolonged *P. fluorescens* bacteraemia observed in outbreaks linked to contaminated intravenous products and indwelling catheters [9,105]. The mechanisms regulating biofilm formation in NAPs likely involve complex signaling pathways similar to those in *P. aeruginosa*, adapting their sessile lifestyle to specific environmental cues.

The remarkable metabolic versatility inherent to the *Pseudomonas* genus [106] is a crucial factor in the opportunistic pathogenicity of NAPs. Their ability to utilize a wide range of carbon and energy sources allows them to colonize diverse and often nutrient-limited host niches, such as the respiratory tract, urinary tract, or wounds. Adaptation to specific host environments involves metabolic rewiring to utilize available substrates (e.g., amino acids, lipids in lung mucus for respiratory pathogens) and cope with host-imposed stresses (e.g., oxidative stress, nutrient limitation) [107]. This metabolic flexibility, honed in diverse environmental settings, provides NAPs with the foundational tools to survive and persist within the human body when host defences are compromised. For instance, the unique amino acid metabolism genes found enriched in clinical *P. putida* isolates may reflect adaptation to nutrient availability within host tissues [43].

### 3.4. Establishing Genotype–Phenotype Links in NAP Pathogenesis

While genomic sequencing provides a blueprint of potential capabilities, establishing definitive links between specific genotypes and virulence-related phenotypes in NAPs requires rigorous experimental validation.

Approaches successfully used in *P. aeruginosa*, such as large-scale mutant library screening for diverse phenotypes (phenomics) [108,109,110], transcriptomics/proteomics under host-relevant conditions, targeted gene deletions and complementation, and the use of relevant infection models (in vitro cell culture, animal models), are essential for NAPs. For example, studies in *P. aeruginosa* have linked mutations in quorum-sensing regulators like *lasR* to specific changes in virulence factor production and antibiotic resistance phenotypes [111,112,113]. Similar studies are needed for NAPs to:

- Confirm the function of putative virulence genes identified through homology searches (e.g., T3SS effectors in *P. fluorescens*).
- Determine the contribution of specific metabolic pathways to survival and growth in host environments (e.g., siderophore systems in clinical *P. putida*) [114].
- Elucidate the regulatory networks controlling virulence gene expression in response to host cues.
- Validate the role of genes identified in genomic comparisons between clinical and environmental strains (e.g., stress resistance genes in clinical *P. putida*) [115].

Generating robust genotype–phenotype correlations is critical for moving beyond descriptive genomics towards a predictive understanding of NAP pathogenesis and for identifying novel therapeutic targets.

### 3.5. Interspecies Competition in Polymicrobial Infections Involving NAP

NAP infections frequently occur in polymicrobial settings, such as chronic wounds, the cystic fibrosis lung, or device-associated infections, where they coexist and interact with other bacteria and fungi [116,117]. These interspecies interactions can significantly influence the behaviour, virulence, and antibiotic susceptibility of the involved organisms. Studies involving *P. aeruginosa* have shown that interactions with organisms like *Candida albicans* or other bacteria can trigger changes in virulence factor production (e.g., pyocyanin, rhamnolipids) and alter susceptibility to antibiotics. Competition for essential nutrients like iron can be a major driver of these interactions [118,119]. Given that NAPs possess similar interaction tools (e.g., T6SS for bacterial warfare, siderophores for iron competition) [51,52], it is highly likely that their virulence and response to treatment in vivo are modulated by the surrounding microbial community.

## 4. Diagnostic Challenges and Advances for Non-Aeruginosa *Pseudomonas* Infections

Accurate and timely identification of the causative agent is fundamental for managing bacterial infections. However, diagnosing infections caused by NAP species presents unique challenges, largely stemming from their taxonomic complexity and phenotypic similarity to other Gram-negative non-fermenters, including *P. aeruginosa* [29].

### 4.1. Traditional Diagnostic Methods and Their Limitations

Conventional methods used in clinical microbiology laboratories for bacterial identification rely on culture followed by phenotypic characterization and biochemical testing. While *Pseudomonas* species are generally readily culturable on standard laboratory media, differentiating NAPs from *P. aeruginosa* and from each other based solely on traditional tests can be unreliable [67,77].

Culture Characteristics: While some traits like pigment production (pyocyanin/pyoverdin) and growth at 42 °C are characteristic of *P. aeruginosa*, NAPs typically lack these specific features. *P. fluorescens* may produce fluorescein (pyoverdine) but does not grow well at 42 °C. *P. putida* is non-fluorescent and does not grow at 42 °C. *P. stutzeri* is non-fluorescent and may exhibit characteristic dry, wrinkled colonies, but colony morphology can be variable [67].

Biochemical Testing: Commercial systems employing panels of biochemical tests (e.g., API strips, automated systems like Vitek) are widely used for bacterial identification. However, these systems often struggle to accurately differentiate closely related NAP species within complexes like *P. fluorescens* or *P. putida*. Databases may lack comprehensive profiles for less common NAP species, leading to results like “low discrimination,” “inconclusive,” “unidentified,” or broad categorizations such as “various nonfermenting gram-negative bacilli”. These limitations mean that NAP species are frequently misidentified or reported simply as “*Pseudomonas* spp.” in clinical settings [67,77].

### 4.2. Misidentification of NAP Species: Frequency and Common Pitfalls

The unreliability of traditional methods for NAP identification is increasingly evident. Studies employing definitive genomic methods have revealed high rates of misidentification for isolates previously identified by biochemical systems. One study re-evaluating clinical isolates originally identified as *P. putida* or *P. fluorescens* using WGS found that most were misidentified, belonging to various other known or even novel *Pseudomonas* species [5,68].

Common pitfalls include:

- Confusion among NAP species: Closely related species within the *P. fluorescens*, *P. putida*, or *P. stutzeri* complexes are difficult to resolve biochemically [68,120].
- Confusion with *P. aeruginosa*: While some key tests differentiate typical strains, atypical strains might be misclassified [121].
- Confusion with other non-fermenting Gram-negative rods: Genera like *Stenotrophomonas*, *Acinetobacter*, or *Burkholderia* can sometimes present diagnostic challenges [122].

This frequent misidentification significantly hinders our understanding of the true epidemiology, clinical spectrum, and antimicrobial susceptibility patterns of individual NAP species, potentially impacting patient management and infection control efforts.

### 4.3. Modern Diagnostic Approaches

Advances in molecular and proteomic techniques offer more accurate and rapid alternatives for NAP identification:

MALDI-TOF MS: This technology identifies microorganisms based on their unique protein profiles, primarily ribosomal proteins. It is rapid (minutes from a colony), cost-effective, and has revolutionized routine bacterial identification in many clinical laboratories. MALDI-TOF MS can accurately identify many *Pseudomonas* species, including common NAPs, provided comprehensive and well-curated reference databases are available. However, its ability to differentiate very closely related species or novel species within complexes may still be limited by database coverage and inherent spectral similarity. It can potentially complement WGS by providing rapid initial identification [69].

16S rRNA Gene Sequencing: While a cornerstone of bacterial phylogeny, sequencing the full 16S rRNA gene often lacks sufficient resolution for reliable species-level identification within the *Pseudomonas* genus, particularly for distinguishing members of species complexes. Its utility is improved when combined with sequencing of additional, more variable housekeeping genes (an MLST-like approach) [69].

Species-Specific PCR: Polymerase chain reaction assays targeting unique gene sequences can provide rapid and sensitive detection of specific NAP species. However, designing highly specific primers that account for intraspecific variation and avoid cross-reactivity with closely related species is challenging, especially given the genomic plasticity of *Pseudomonas* spp. Multiplex PCR assays targeting several NAPs simultaneously could be developed but require careful validation. Isothermal amplification methods (e.g., LAMP) offer potential for rapid, point-of-care detection if species-specific targets can be reliably identified [123,124,125].

WGS in Clinical Diagnostics: WGS provides the highest resolution for bacterial identification and characterization. It allows for definitive species identification based on whole-genome comparisons (e.g., ANI, dDDH), resolving taxonomic ambiguities within species complexes. Furthermore, WGS data can simultaneously provide information on phylogenetic relationships (for outbreak tracking), predict antimicrobial resistance mechanisms, and identify virulence factors. While currently more expensive and time-consuming than MALDI-TOF, the decreasing costs and improving bioinformatics pipelines are making WGS increasingly feasible for reference laboratories and potentially, in the future, for more routine clinical diagnostics, especially for problematic or critical isolates [70].

### 4.4. Towards Standardized Identification Protocols for NAP

Given the limitations of traditional methods and the varying capabilities of newer technologies, a standardized approach to NAP identification in clinical laboratories is needed [126]. A tiered strategy could balance accuracy, speed, and cost:

MALDI-TOF MS should be the primary method for rapid identification of isolates resembling *Pseudomonas*. Reliable species-level identification can often be achieved for common NAPs if databases are robust [5,71,72].

For isolates where MALDI-TOF yields ambiguous results (e.g., low confidence score, identification only to genus/group level), or for isolates from critical sterile sites (blood, CSF), outbreaks, or those exhibiting unusual resistance patterns, further characterization is warranted.

WGS is emerging as the definitive method for resolving complex cases, identifying novel species, and providing comprehensive genomic context (including AMR and virulence prediction) [73,74]. WGS should be considered the reference standard, particularly for epidemiological studies and research purposes. Crucial to the success of any protocol is the continuous improvement and curation of databases for both MALDI-TOF MS spectral profiles and genomic sequences, ensuring they encompass the known diversity within NAP species complexes [5,69]. Collaboration between clinical laboratories and research institutions is vital for validating methods and expanding reference databases. Table 2 compares diagnostic methods for NAP.

## 5. Therapeutic Strategies for NAP Infections: Current and Future Perspectives

Treating infections caused by NAP species is complicated by several factors, including the inherent difficulties in accurate species identification, the lack of species-specific antimicrobial susceptibility testing (AST) breakpoints and clinical guidelines, and the increasing prevalence of AMR [5,127,128].

### 5.1. Historical Overview of Antimicrobial Treatment for NAP

Historically, due to the challenges in differentiating NAPs from the more common *P. aeruginosa*, infections caused by NAPs were often treated empirically using regimens designed for *P. aeruginosa* [129]. Treatment decisions were typically based on the susceptibility profile of the isolate, interpreted using *P. aeruginosa* breakpoints, if available, or general non-fermenter guidelines [130]. Early reports noted variable susceptibility but often sensitivity to agents like polymyxins and gentamicin. The choice of antibiotics was largely guided by in vitro data from the specific isolate, often involving combination therapy, particularly for serious infections, mirroring practices for *P. aeruginosa*. However, the true efficacy of these approaches specifically against various NAP species remained poorly defined due to the confounding factor of misidentification.

### 5.2. Current Antibiotic Arsenal and Susceptibility Patterns in NAP

The activity of standard antibiotics with known anti-*P. aeruginosa* activity against NAP species is variable and often unpredictable. Susceptibility must be determined on a case-by-case basis through in vitro testing.

- β-Lactams: Agents like piperacillin–tazobactam, ceftazidime, and cefepime may show activity against some NAP isolates, but resistance is common. Carbapenems (imipenem, meropenem) were historically more reliable but resistance, often mediated by carbapenemases, is a growing concern in species like *P. putida* and *P. stutzeri*. Aztreonam’s activity is generally limited to aerobic Gram-negatives but can be an option if susceptible [131,132,133].
- Aminoglycosides: Gentamicin, tobramycin, and amikacin can be effective against some NAPs, but resistance mechanisms exist. They are often used in combination therapy for serious infections [133].
- Fluoroquinolones: Ciprofloxacin and levofloxacin show variable activity. While some studies report good susceptibility (e.g., >85% in NAPs from cancer patients), resistance is also documented [133].
- Polymyxins: Colistin remains active against many Gram-negatives, including *P. aeruginosa*, but its use is limited by toxicity. Resistance in NAPs has been reported [134].

A major challenge is the lack of established, species-specific clinical breakpoints for NAPs in guidelines from organizations like EUCAST or CLSI [135,136]. Laboratories typically rely on breakpoints established for *Pseudomonas aeruginosa* or “*Pseudomonas* spp.”. This extrapolation is problematic, as the pharmacokinetic/pharmacodynamic (PK/PD) relationships and intrinsic susceptibility levels may differ between NAP species and *P. aeruginosa*, potentially leading to inappropriate treatment decisions.

NAP species possess both intrinsic and acquired mechanisms of resistance, similar to *P. aeruginosa*.

- Intrinsic Resistance: This includes low outer membrane permeability, which restricts antibiotic entry, and the presence of chromosomally encoded efflux pumps (e.g., RND family pumps) that actively expel various antibiotics from the cell. Some species may also possess chromosomal β-lactamases (e.g., AmpC-like enzymes) [128,137].
- Acquired Resistance: NAPs readily acquire resistance genes via HGT, primarily through plasmids, transposons, and integrons. Key acquired mechanisms include:
  ∘ β-Lactamase Production: This is a major mechanism conferring resistance to β-lactam antibiotics. NAPs can acquire genes encoding various enzymes, including Extended-Spectrum β-Lactamases (ESBLs) and, critically, carbapenemases. Metallo-β-lactamases (MBLs), particularly of the VIM and IMP types, are a significant concern in NAPs like *P. putida* and *P. stutzeri*, as they hydrolyze nearly all β-lactams, including carbapenems. Other carbapenemases (e.g., KPC, NDM, OXA-type) found in *P. aeruginosa* could potentially spread to NAPs. A novel carbapenemase, *blaPFM-2*, was identified in an Antarctic *P. fluorescens* isolate [54].
  ∘ Efflux Pump Upregulation: Mutations leading to overexpression of intrinsic efflux pumps can confer resistance to multiple antibiotic classes.
  ∘ Target Site Modification: Mutations in genes encoding antibiotic targets (e.g., *gyrA*/*parC* for fluoroquinolones, ribosomal modifications for aminoglycosides) can reduce antibiotic binding and efficacy.
  ∘ Porin Loss: Decreased expression or mutation of outer membrane porins can reduce the influx of certain antibiotics, particularly carbapenems.

The presence of MDR strains, particularly those producing carbapenemases like MBLs, poses a significant therapeutic challenge for NAP infections. 

### 5.3. Novel Antibiotics and Therapeutic Agents for Multidrug-Resistant (MDR)-NAP

The emergence of MDR NAPs necessitates exploring newer antimicrobial agents, many developed primarily targeting MDR *P. aeruginosa* and other difficult-to-treat Gram-negatives. In vitro data suggest potential utility against NAPs, but clinical data specifically for NAP infections are scarce.

- Cefiderocol: A novel siderophore cephalosporin that utilizes bacterial iron uptake systems to enter the periplasmic space, bypassing some common resistance mechanisms like porin loss and efflux. It exhibits broad in vitro activity against Gram-negatives, including carbapenem-resistant isolates. Studies show high in vitro activity against NAP clinical isolates, including MBL-producing *P. putida* and *P. stutzeri* strains. Susceptibility rates often exceed 90% based on *P. aeruginosa* breakpoints. Clinical and preclinical data support its role as an option for difficult-to-treat *P. aeruginosa*, potentially extending to NAPs, especially MBL producers where other novel agents fail [138,139,140,141,142,143].
- Ceftazidime/avibactam (CZA): Combines ceftazidime with a novel β-lactamase inhibitor, avibactam, which inhibits Ambler class A (including ESBLs, KPC), class C (AmpC), and some class D (e.g., OXA-48) β-lactamases. It is generally not active against MBLs (class B). In vitro studies show variable activity against NAPs; it may be effective against non-MBL-producing MDR strains but is unlikely to be useful for VIM- or IMP-producing isolates commonly found in resistant NAPs [139,142,143,144].
- Ceftolozane/tazobactam (C/T): Combines an advanced-generation cephalosporin, ceftolozane, with the established inhibitor tazobactam. Ceftolozane has enhanced stability against AmpC and some efflux pumps compared to older cephalosporins. Tazobactam inhibits many class A β-lactamases but not AmpC or carbapenemases. C/T shows good activity against many *P. aeruginosa* isolates but, like CZA, is not active against MBLs. Its utility against MDR NAPs is likely limited to non-MBL producers. Resistance can emerge during therapy [142,145].
- Other Novel Agents/Approaches: Agents like imipenem–relebactam (carbapenem + novel inhibitor active against KPC/AmpC) or meropenem–vaborbactam (active against KPC) may have roles depending on the specific resistance mechanisms present, but MBLs remain a major challenge for most current β-lactam-based therapies [146]. Alternative strategies explored for *P. aeruginosa*, such as phage therapy [147,148,149], antimicrobial peptides [150,151], or quorum sensing inhibitors [152,153], could potentially be adapted for NAP infections but require significant further research. Combination therapy using novel agents or combining novel and older agents based on synergy testing might be necessary for highly resistant strains [142,154,155].

It is imperative that susceptibility testing is performed for novel agents against NAP isolates, interpreting results cautiously using available (often *P. aeruginosa*) breakpoints until NAP-specific criteria are established.

### 5.4. Challenges in Treating NAP Infections and Future Directions

Managing NAP infections effectively faces several hurdles:

1. Diagnostic Delays/Inaccuracy: Misidentification can lead to delayed or inappropriate therapy [5,67].
2. Lack of Clinical Data: There is a paucity of clinical trials specifically evaluating antibiotic efficacy for NAP infections. Treatment decisions often rely on extrapolation from *P. aeruginosa* data or in vitro results alone [6].
3. Breakpoint Uncertainty: Using *P. aeruginosa* breakpoints for NAPs may not accurately predict clinical outcomes [156,157].
4. Emerging Resistance: The potential for NAPs to harbor or acquire significant resistance mechanisms, including carbapenemases, limits therapeutic options [133,158].

Future research should prioritize:

- Developing and validating rapid and accurate diagnostic methods specifically for clinically relevant NAP species.
- Establishing species-specific AST breakpoints for NAPs through integrated PK/PD and clinical outcome studies.
- Conducting clinical trials evaluating the efficacy of existing and novel antibiotics specifically for infections caused by common NAP species (*P. fluorescens*, *P. putida*, *P. stutzeri*).
- Enhancing surveillance for AMR in NAP species in both clinical and environmental settings.
- Investigating alternative therapeutic strategies, including phage therapy and virulence factor inhibitors, for MDR NAP infections.

## 6. Environmental and Biotechnological Roles of Non-Aeruginosa *Pseudomonas*

Beyond their clinical relevance, NAP species are renowned for their metabolic versatility and robustness, making them key players in various environmental processes and attractive platforms for biotechnology.

### 6.1. NAP Species as Biocatalysts and Bioremediation Agents

The ability of many NAPs to thrive in diverse, sometimes harsh, environments is linked to their capacity to metabolize a wide array of organic compounds, including pollutants and xenobiotics. This metabolic prowess is actively exploited:

- *P. putida*: This species, particularly strain KT2440, is a workhorse in bioremediation and biotechnology. Its diverse catabolic pathways allow it to degrade aromatic hydrocarbons (e.g., toluene, benzene), solvents, naphthalene, and other pollutants, making it useful for cleaning contaminated soil and water [61,62,159]. Strain KT2440, often engineered with plasmids like the TOL plasmid, serves as a model for biodegradation studies. *P. putida* is also engineered for biocatalysis, producing valuable chemicals like short-chain ketones, bioplastics (polyhydroxyalkanoates, PHAs) from waste materials like styrene, rhamnolipids (biosurfactants), terpenoids, polyketides, and amino acid-derived compounds [160]. Its tolerance to organic solvents and amenability to genetic manipulation (using tools reviewed by Nikel et al., 2016) are key advantages [161,162].
- *P. fluorescens* complex: Members of this group are widely studied as plant growth-promoting rhizobacteria (PGPR) and biocontrol agents. They suppress plant pathogens through mechanisms like competition for nutrients (e.g., iron via siderophores), production of antifungal/antibacterial secondary metabolites (e.g., DAPG, phenazines, pyrrolnitrin), and induction of systemic resistance in the host plant. Several *P. fluorescens*-based biocontrol products have been commercialized for agricultural use [63,64].
- *P. stutzeri* complex: Known for its potent denitrification capabilities, *P. stutzeri* plays a significant role in nitrogen cycling in soil and aquatic environments and is a model organism for studying this process [10]. Its metabolic versatility also extends to degrading pollutants, and it shows potential as a host for recombinant protein production, including membrane proteins [163,164].

The inherent metabolic capabilities and stress resistance of NAPs make them valuable tools for addressing environmental pollution and developing sustainable bioproduction processes.

### 6.2. Biosafety Considerations for Biotechnological Applications

The use of NAPs, particularly genetically engineered microbes (GEMs), in large-scale industrial processes or for environmental release necessitates careful biosafety assessment and management [65,66].

While wild-type strains like *P. putida* KT2440 are generally considered safe (Biosafety Level 1, BSL-1) and non-pathogenic to healthy humans or the environment under normal conditions [78], several factors require consideration for biotechnological applications:

- Opportunistic Pathogenicity: As discussed, NAPs can cause infections in vulnerable individuals. Large-scale cultivation or release increases potential exposure levels. Furthermore, genetic modifications aimed at enhancing biotechnological traits could inadvertently affect virulence or fitness [5].
- Horizontal Gene Transfer: A primary concern is the potential transfer of engineered genetic material (e.g., novel metabolic pathways, selectable markers like antibiotic resistance genes) from the released GEM to the indigenous microbiota. Transfer of ARGs used in constructing engineered strains is particularly problematic, potentially contributing to the environmental pool of resistance [75,84].
- Ecological Impact: Introducing large populations of engineered NAPs could potentially disrupt native microbial community structures or ecological processes, although evidence for significant adverse effects from wild-type *P. putida* at the population level is limited [65].

Risk assessments, like those conducted by regulatory agencies (e.g., US EPA, Health Canada) for environmental release applications of engineered *P. putida*, evaluate factors such as the nature of the genetic modification, the characteristics of the host strain, the potential for survival and dissemination, the likelihood and consequences of HGT, and potential effects on non-target organisms and human health [66]. Even for BSL-1 organisms, modifications like the introduction of widely used clinical antibiotic resistance markers raise concerns for larger-scale applications [84].

Historically, biosafety frameworks for GEMs emphasized strict physical and biological containment to prevent their escape into the environment. Physical containment involves specialized facilities (labs, greenhouses), while biological containment employs genetic modifications to limit the GEM’s survival or gene transfer capabilities outside the intended environment (e.g., auxotrophy, kill switches) [58].

However, for many environmental applications (e.g., in situ bioremediation, agricultural inoculation), complete containment is impractical or defeats the purpose [65,66]. Furthermore, biological containment systems have limitations and may not always be fully effective. This has led to an evolution in biosafety paradigms, acknowledging that absolute containment is often unattainable for environmental releases. Consequently, there is a growing emphasis on robust risk assessment prior to release, coupled with effective monitoring strategies to track the fate and impact of GEMs post-release [66,165,166]. This shift focuses on understanding and managing potential risks rather than solely relying on preventing escape. Modern synthetic biology tools are also enabling the design of more sophisticated biological containment systems, such as inducible kill switches based on CRISPR-Cas9 targeting essential or repetitive genomic regions or genome recoding strategies that create genetic isolation, enhancing biosafety for engineered NAPs like *P. putida* [58,167].

Effective monitoring is crucial for assessing the persistence, spread, and potential ecological or health impacts of released engineered NAPs. Recent developments leverage advances in molecular biology:

- Molecular Markers: Engineered strains can be tagged with unique DNA sequences (barcodes) or fluorescent reporter genes (e.g., GFP) that allow for specific detection and tracking against the background of native bacteria [166,168].
- Quantitative PCR (qPCR): Highly sensitive qPCR assays can be designed to detect and quantify the specific DNA markers of the released GEM in environmental samples (soil, water) [168].
- Sequencing-Based Methods: Metagenomic sequencing of environmental DNA can provide a broader picture of the microbial community and detect the presence and relative abundance of the engineered NAP. Targeted amplicon sequencing or WGS of isolates recovered from the environment can confirm identity and track genetic changes post-release [75].
- Biosensors: Whole-cell biosensors, potentially using the engineered NAP itself or other reporter organisms, could be developed to detect specific signals related to the GEM’s presence or activity in situ [65].

The development and application of these monitoring tools are essential components of modern biosafety frameworks for the responsible use of engineered NAPs in biotechnology and environmental management.

### 6.3. Impact of Large-Scale Environmental Use of NAP on Human Health

A critical question arising from the dual nature of NAPs is whether their large-scale use in agriculture (biocontrol, PGPR) or bioremediation could inadvertently increase the risk of human infections, either through direct exposure to the applied strains or by facilitating the spread of AMR genes into clinical pathogens. At present these concerns are theoretical: they are biologically plausible but, as detailed below, are not supported by direct evidence of such effects occurring in practice.

Currently, there appears to be limited direct evidence from systematic studies or documented cases definitively linking the large-scale environmental application of specific, well-characterized NAP strains (like *P. fluorescens* biocontrol agents or *P. putida* bioremediation strains) to subsequent outbreaks or increased incidence of human infections caused by those same strains. Wild-type strains used in many applications are generally considered low risk (BSL-1) [65,79].

However, several factors warrant ongoing vigilance and research:

- Opportunistic Potential: Even BSL-1 strains can cause infections in highly susceptible individuals, and increased environmental prevalence could theoretically increase exposure risk [67].
- AMR Spread: The environment is a recognized reservoir of ARGs. Large-scale release of NAPs, especially if they carry ARGs or acquire them from the environment, could contribute to the dissemination and potential transfer of these genes to human pathogens.
- Strain Characterization: Ensuring that strains intended for environmental release are thoroughly characterized, confirmed as non-pathogenic variants, and free from undesirable ARGs is crucial [65,79].
- Data Gaps: There is a lack of long-term surveillance specifically designed to track potential links between environmental NAP applications and clinical infection rates [65].

While widespread negative health impacts from current environmental uses of NAPs have not been clearly demonstrated, the potential for AMR spread and opportunistic infections necessitates continued monitoring, careful strain selection and engineering practices, and adherence to regulatory oversight.

## 7. Future Perspectives and Integrated Approaches

The study of NAP species sits at a fascinating intersection of clinical microbiology, environmental science, and biotechnology. While significant progress has been made, particularly driven by genomic technologies, numerous knowledge gaps and challenges remain. Addressing these requires integrated approaches and focused research efforts.

### 7.1. The One Health Framework for Understanding NAP

Given their ubiquity in the environment, presence in animals, potential use in agriculture and industry, and role as human opportunistic pathogens, NAP species exemplify the interconnectedness of human, animal, and environmental health. A One Health approach is therefore essential for a holistic understanding of these organisms. This framework encourages collaboration across disciplines to:

- Track the flow of NAPs and associated genetic elements (especially ARGs) between different reservoirs (soil, water, plants, animals, humans, clinical settings) [79].
- Assess the impact of human activities (e.g., agriculture, industrial discharge, antibiotic use in medicine and farming) on the evolution and dissemination of resistance and virulence in environmental and clinical NAP populations [65,66].
- Understand the potential risks associated with using NAPs in environmental applications (bioremediation, biocontrol) on human and animal health [65].
- Develop integrated strategies for surveillance, prevention, and control of both NAP infections and the spread of AMR that consider all facets of their ecology.

By adopting a One Health perspective, researchers, clinicians, public health officials, and policymakers can better address the complex challenges and opportunities presented by the multifaceted NAP species. Continued research integrating genomic, ecological, clinical, and biotechnological insights is crucial for navigating the dual roles of these remarkable bacteria.

### 7.2. Emerging Technological and Translational Directions

Beyond the conceptual One Health framing above, several concrete avenues merit dedicated investment. Real-time long-read sequencing (e.g., Oxford Nanopore) is increasingly affordable and portable, allowing near-real-time species confirmation and resistance-gene detection from isolates and, in some settings, from primary specimens. Embedding such workflows in antimicrobial stewardship programmes could shorten the diagnostic delay that currently drives empirical, *P. aeruginosa*-oriented therapy for NAP infections, and could flag carbapenemase carriage (e.g., *blaVIM*) before phenotypic susceptibility testing is complete.

Precision antimicrobials offer a second avenue. Sequence-specific agents that deliver CRISPR-Cas systems via phage or conjugative vectors to cleave resistance genes or essential loci could, in principle, target multidrug-resistant NAPs while sparing commensal flora; their development for non-aeruginosa species is at an early stage and will require NAP-specific delivery vehicles and validated genomic targets.

Finally, for the environmental and industrial strains discussed in Section 6, synthetic-biology containment—inducible kill switches, synthetic auxotrophies, and genome-recoding strategies that create genetic isolation—provides a proactive means of limiting HGT of engineered or resistance-associated genetic material. Coupling such safeguards with the molecular monitoring tools described in Section 6.2 would operationalise the shift from containment-only to risk-managed deployment.

## Figures and Tables

**Table 1:** Comparative Properties of Key Non-Aeruginosa *Pseudomonas* Species Complexes.

Feature	*P. fluorescens* Complex	*P. putida* Group	*P. stutzeri* Complex
Key Phenotypic Traits [3,10]	- Fluorescent pigment production (pyoverdine) - Generally psychrotolerant (growth at 4 °C) - Obligate aerobe (some use nitrate) - Oxidase positive - Does not typically grow at 42 °C	- Fluorescent - Mesophilic (optimal ~30 °C) - Oxidase positive - Does not typically grow at 42 °C - Metabolically versatile (e.g., degrades solvents)	- Non-fluorescent - Denitrification common - Mesophilic (optimal ~35 °C, range 4–44 °C) - Oxidase positive - Often wrinkled/dry colonies - Natural transformation common in many strains
Common Clinical Manifestations [3,6,8,9,10]	- Bacteremia (often iatrogenic: contaminated blood products, IV fluids/equipment) - Respiratory isolates (significance often unclear) - Urinary tract infections - Association with Crohn’s disease (seroreactivity)	- Bacteremia (often catheter-related or in immunocompromised) - Skin & Soft Tissue Infections (SSTIs, esp. post-trauma, burns, chronic wounds) - Urinary tract infections - Pneumonia - Rare cause of cellulitis	- Wound infections - Urinary tract infections - Bacteremia (often in patients with comorbidities) - Ear infections (otitis) - Pneumonia, meningitis, endocarditis (rare)
Known Virulence Factors [3,10]	- Biofilm formation - Siderophores (pyoverdine) - Hemolysins/Phospholipase C (some strains) - Bioactive secondary metabolites (e.g., DAPG, phenazines in specific subgroups)	- Biofilm formation - Siderophores (pyoverdine) - Adhesins, Motility (flagella) - Potential lipases, proteases - Toxin/Antitoxin systems (in some clinical isolates)	- Motility (flagella) - Denitrification enzymes (role in niche adaptation) - Potential siderophores - Limited data on specific toxins/enzymes directly linked to human virulence
Type III Secretion (T3SS) [51]	- Present in some strains/subclades - Linked to hemolytic activity & macrophage interactions in some isolates - Effectors identified (e.g., RopAA, RopM, RopB in Q8r1-96)	- T3SS-like cluster identified in at least one clinical isolate - Role in human infection unclear/understudied	- Generally considered absent or not well-characterized in the context of human infection
Type VI Secretion (T6SS) [51]	- Present (multiple clusters possible) - Likely role in interbacterial competition	- Present (e.g., 3 clusters in KT2440) - Demonstrated role in antibacterial activity against competitors (incl. phytopathogens) - Role in human infection unknown	- Present in some strains - Role in human infection context not established
Genetic Tractability [39,40,41,42,43,44,45,57,58]	- Moderate; Transformation possible (electroporation) - Phage-derived recombinases used - Plasmid vectors available	- High; Well-established tools - Electroporation, conjugation effective - Plasmids (e.g., pBBR1, pSEVA), inducible promoters available - Recombineering, CRISPR-Cas9 systems developed	- Moderate; Natural transformation competence in many strains - Electroporation possible - Broad-host-range vectors functional (e.g., pBBR1-based, pL2020) - Genetic inverter device demonstrated
Key Biotechnological Apps [21,22,59,60,61,62,63,64,65,66]	- Biocontrol (plant pathogens) - Plant Growth Promotion (PGPR) - Bioremediation (potential)	- Bioremediation (hydrocarbons, solvents, pollutants) - Industrial biocatalysis (chemical synthesis) - Bioplastic (PHA) production - Biocontrol	- Denitrification studies (model organism) - Bioremediation (pollutants) - Potential host for membrane protein production
Recommended ID Methods [5,27,28,29,30,31,35,67,68,69,70,71,72,73,74,75]	- MALDI-TOF MS (database dependent) - WGS (gold standard) - MLST (phylogeny) - 16S rRNA + housekeeping genes	- MALDI-TOF MS (database dependent) - WGS (gold standard) - MLST (phylogeny) - 16S rRNA + housekeeping genes	- MALDI-TOF MS - WGS (definitive) - MLST (phylogeny) - 16S rRNA + housekeeping genes - Colony morphology (wrinkled) suggestive
Common Misidentification [5,28,67,68,76,77]	- Often misidentified by biochemical systems - Confused with other *Pseudomonas* spp. or non-fermenters	- Often misidentified by biochemical systems - Confused with other *Pseudomonas* spp. or non-fermenters	- Historically confused with other non-fluorescent *Pseudomonas* (e.g., *P. mendocina*) before genomic methods - Biochemical systems may struggle
Biosafety Level (BSL) [65,66,78,79]	- Generally BSL-1 (wild-type)	- Generally BSL-1 (wild-type, e.g., KT2440)	- Generally BSL-1 (wild-type)
Key Risk Assessment Needs [4,9,65,66,75,80,81,82,83,84]	- Potential for opportunistic infections (esp. engineered strains or high exposure) - HGT of engineered/resistance genes - Contamination of medical products	- Potential for opportunistic infections (esp. MDR strains, engineered strains, high exposure) - HGT of engineered/resistance genes (e.g., AMR markers) - Contamination of medical products (e.g., blood)	- Potential for opportunistic infections (esp. in compromised hosts) - HGT of resistance genes (e.g., *blaVIM-2* plasmid)

**Table 2:** Comparison of Diagnostic Methods for Non-Aeruginosa *Pseudomonas* Species.

Method	Principle	Speed (From Colony)	Cost (Relative)	Accuracy for NAP Species	Key Advantages	Key Limitations
Traditional Culture & Biochemical [5,67,68,77,120,121,122]	Growth characteristics, enzymatic reactions	24–72+ h	Low	Often poor for species complexes; High misidentification rate	Widely available, inexpensive	Slow, labor-intensive, poor resolution for NAPs, unreliable
MALDI-TOF MS [69,71,72]	Analysis of protein mass spectra (mainly ribosomal proteins)	Minutes	Low-Moderate	Good for common species; Database dependent	Rapid, cost-effective per test, high throughput	Requires specific instrument, database limitations for rare/novel/closely related species
16S rRNA Sequencing [69]	Sequencing of conserved ribosomal RNA gene	12–48 h	Moderate	Limited for species-level resolution in *Pseudomonas*	Universal target, established phylogenetic marker	Poor discrimination between closely related species/complexes
MLST (Housekeeping Gene Sequencing) [69]	Sequencing of multiple conserved housekeeping genes	24–72 h	Moderate-High	Good phylogenetic resolution; Better than 16S rRNA	Standardized schemes available for some groups, good for epidemiology	More complex than 16S, may not resolve all species ambiguities
Species-Specific PCR [123,124,125]	Amplification of unique gene targets	2–6 h	Moderate	Potentially high if target is truly specific & conserved	Rapid, sensitive, potential for direct detection	Requires validated species-specific targets (difficult for diverse NAPs), potential for false +/−
WGS (Whole Genome Sequencing) [70,73,74]	Sequencing the entire bacterial genome	Days (incl. analysis)	High	Gold standard; highest accuracy and resolution	Definitive ID, AMR/virulence prediction, outbreak analysis, novel species discovery	Higher cost, longer turnaround time, requires bioinformatics expertise

## Data Availability

No new data were created or analyzed in this study. Data sharing is not applicable to this article.
